# Polysaccharide from *Hemerocallis citrina* Borani by subcritical water with different temperatures and investigation of its physicochemical properties and antioxidant activity

**DOI:** 10.3389/fnut.2022.982695

**Published:** 2022-08-11

**Authors:** Yongrui Ti, Yanli Zhang, Yüqian Ban, Xiaoxiao Wang, Yüqing Hou, Zihan Song

**Affiliations:** Institute of Vegetables and Flowers, Chinese Academy of Agricultural Sciences, Beijing, China

**Keywords:** *Hemerocallis citrina* Borani, subcritical water extraction, polysaccharide, physicochemical properties, antioxidant activity

## Abstract

*Hemerocallis citrina* Borani as a low-cost vegetable, has various health benefits. However, the industry of *H. citrina* Borani is in the state of primary processing, with poor economic benefits. This study aimed to investigate the physicochemical properties, and the antioxidant activity of *H. citrina* Borani polysaccharide (HCBP) using subcritical water extraction (SWE) at different temperatures, to expand the value of *H. citrina* Borani. HCBP mainly composed of nine monosaccharides (glucose, galactose, rhamnose, fucose, mannose, arabinose, xylose, galacturonic acid, and glucuronic acid), among which the content of neutral sugar was higher and uronic acid was lower. HCBP contained glycosidic bond of β-configurations and trace quantities protein. The molecular weight of HCBP decreased with increasing temperature. Shear thinning occurred in HCBP with the increase of shear rate (0.01–1 s^−1^), and the apparent viscosity of HCBP decreased at higher temperature (150–160°C) with the increase continuously of shear rate (1–10 s^−1^), but almost remained constant at lower temperature (130–140°C). Scanning electron microscope showed that HCBP had rough surface, loose structure, obvious particle gap, and irregular shape. In addition, HCBP extracted at 160°C had strong FRAP activity, and HCBP extracted at 130 and 140°C had better ABTS radical scavenging activity. This study suggests that HCBP extracted by SWE could provide a cheap raw material as food thickening agent and natural antioxidants.

## Introduction

*Hemerocallis citrina* Borani is perennial herbs in the Liliaceae family, commonly known as daylily, and its flower buds are one of the most commonly consumed vegetables in Asian countries including China, Japan, and Korea (1). *Hemerocallis citrina* Borani is rich in nutrition, which is a typical health vegetable with rich in protein, vitamins, and minerals. *H. citrina* Borani has been demonstrated multiple functions including antioxidant (2), antidepressant (3, 4), lactation improving (5), and sleep promoting (6), as documented in the medicinal book “Compendium of Materia Medica” (7). The industry of *H. citrina* Borani is so far in the state of primary processing, with poor economic benefits and low value-added. At present, the studies on the deep processing of *H. citrina* Borani mainly focusing on the functional components including flavonoids and alkaloids (8–10). However, few studies are reported on *H. citrina* Borani polysaccharide (HCBP). Moreover, the preparation process is the crucial point for industrial utilization of HCBP.

Many methods have been applied to extract polysaccharide, such as hot-water extraction (11), acid/base extraction (12), microwaving-assisted extraction (13), and ultrasound-assisted extraction (14). These traditional techniques have the shortcomings of higher extraction time, high-energy consumption, low extracting efficiency and yield, as well as limited polysaccharide purity. Water that remains liquid state at high temperature (100–374°C) and under high pressure is called subcritical water (SW), also known as superheated water and pressurized hot water. SW has lower dielectric constant, lower viscosity, and higher diffusivity, which makes SW an effective solvent for both polar and non-polar compounds with acid-base catalytic characteristics (15). Subcritical water extraction (SWE) is a promising green extraction technology that uses SW as the only extraction solvent. Obviously, during the polysaccharide extraction, no other chemical reagents are added except water, avoiding the environmental pollution problem (16). Remarkably, SWE has the advantages of short extraction time, high extraction efficiency, strong specificity, simple operation, good cost effectiveness, and environmental friendliness, which has been widely used in the extraction of natural products (17).

At present, the research on the extraction of polysaccharide by SWE is mainly in the aspects of structure analysis and biological activity evaluation. However, there are few studies on the effects of SWE temperature on polysaccharide. In addition, the extraction temperature is closely related to the structure properties of polysaccharide, which significantly affect the functional properties of polysaccharide. For example, SWE temperature significantly affects the physicochemical properties and biological activity of polysaccharide, such as monosaccharide composition (18), molecular weight (19), and antioxidant activity (20). Therefore, the extraction temperature of SW possessed crucial effects on the properties of polysaccharide, the appropriate extraction temperature should be selected according to the characteristics of polysaccharide in actual production.

In this study, four HCBP samples were prepared by subcritical water with different extraction temperatures (130, 140, 150, and 160°C). The composition, structure, and antioxidant activity of the four HCBP samples were firstly systematically analyzed. In addition, the study also determined the physicochemical properties of the four HCBP samples by gel filtration chromatography, high performance liquid chromatography, ultraviolet–visible spectroscopy, infrared spectroscopy, scanning electron microscope, and hybrid rheometer. The present study could provide theoretical and data support for the further utilization of HCBP in the fields including medicine, food and cosmetics, as well as expand the value of *H. citrina* Borani.

## Materials and methods

### Sample preparation and chemicals

*Hemerocallis citrina* Borani was obtained from Qingyang, Gansu, China. Standards of glucose, mannose, fucose, rhamnose, xylose, galactose, arabinose, glucuronic acid, ribose, and galacturonic acid were purchased from Shanghai Yuanye Biotechnology (Shanghai, China). Absolute ethanol was purchased from Fuyu Fine Chemical (Tianjin, China). Trifluoroacetic acid, methanol, sodium hydroxide, hydrochloric acid, carbazole, 1-phenyl-3-methyl-5-pyrazolone, 3,5-dinitrosalicylic acid, sulfuric acid, Coomash bright blue, chloroform, potassium bromide, sodium azide, sodium acetate, anthranone, and other reagents were purchased from Solarbio Biotechnology (Beijing, China). ABTS and FRAP assay kits were purchased from Suzhou Keming Biotechnology (Suzhou, China). Distilled water was used in these experiments.

### Subcritical-water extraction

Laboratory-scale SWE had performed using high temperature and pressure reactor. Briefly, *Hemerocallis citrina* Borani powder sample (5 g) and a certain proportion of distilled water (1:10, 1:15, 1:20, 1:25) were placed in a 1 L subcritical reactor at certain temperature (130, 140, 150, and 160°C) and reaction time (5, 10, 15, and 20 min). The *H. citrina* Borani aqueous solution in the reaction kettle was collected and centrifuged at 6,000 rpm for 10 min. Anhydrous ethanol was added to the solution and subsequently precipitated at 4°C for 12 h. The *H. citrina* Borani ethanol solution was centrifuged in a centrifuge at 6,000 rpm for 10 min, the precipitation was collected, and the polysaccharide was cleaned with anhydrous ethanol for three times to obtain the *H. citrina* polysaccharide.

### Chemical composition analysis

The neutral sugar content was determined with anthrone-sulfuric acid method with minor modifications, with using glucose as standard (21). Uronic acid was estimated in a modified carbazole method using D- galacturonic acid as standard (22). Reducing sugar analysis was conducted using the 3,5-dinitrosalicylic acid (DNS) method (16). Protein content was measured using the Bradford method (23). Ash content was determined by high temperature combustion in muffle furnace.

### Gel permeation chromatography

The molecular weight of HCBP was determined by gel permeation chromatography (24), HCBP was dissolved by sodium azide and sodium acetate and loaded into the chromatography system (40 μL), the column was maintained at 30°C and the flow rate of 1 mL/min. The molecular weight of HCBP was calculated by using calibration curves obtained from standard dextran of different molecular weights.

### Monosaccharide composition

The monosaccharide composition of polysaccharide was analyzed by high performance liquid chromatography (HPLC) according to previous research with slight modified (25). Polysaccharide samples (10.0 mg) dissolved in 3 M trifluoroacetic acid (4.0 mL) in a 10 mL ampoule. The ampoule was sealed with an alcohol blowtorch and hydrolyzed for 2 h in an electric oven at 121°C. Then sodium hydroxide solution, PMP methanol solution and lactose internal standard solution were added into polysaccharides hydrolysate and monosaccharides standard for PMP derivatization, and the remaining aqueous phase was filtered by 0.22-μm membrane and analyzed by HPLC. Chromatographic conditions included Agilent ZORBAX Eclipse Plus (C18, 2.1 × 100 mm,1.8 μm, Agilent, USA) capillary column, diode array detector (250 nm detection wavelength), sodium acetate acetonitrile solution (mobile phase A) and acetonitrile (mobile phase B), 0.6 mL/min flow rate and column at 35°C. The monosaccharide standards including fucose, mannose, rhamnose, glucose, galactose, xylose, arabinose, ribose, glucuronic acid, and galacturonic acid were PMP-labeled and determined by HPLC in the same way.

### Spectrum analysis

#### Ultraviolet–visible (UV) spectrometric analysis

The UV spectrum of HCBP samples were determined with ultraviolet–visible spectroscopy (Shimadzu UV-2600, Japan). Briefly, HCBP samples were prepared with distilled water at a concentration of 0.5 mg/ mL, and UV spectrum from 200 to 900 nm were recorded (26).

#### Fourier transform infrared (FT-IR) spectrometric analysis

Fourier transform infrared spectrophotometer (Thermo Nicolet NEXUS870, Massachusetts, USA) was used to determine the infrared spectrum characteristics of HCBP different temperature samples at a wavelength range of 4,000–400 cm^−1^ (27). The specific use of KBr Tablet pressing method, and then performed for infrared spectrometric determination.

### Scanning electron microscope (SEM) analysis

The HCBP samples were fixed on the carrier platform and sprayed with gold powder. The Morphology of HCBP extracted at different extraction temperature was observed by SEM (Hitachi S-4800, Tokyo, Japan) under an acceleration voltage of 10 kV with different magnifications (5-, 10-, 50-, and 100-μm) (28).

### Rheological analysis

Rheological properties of HCBP samples (2 g/100 mL) were evaluated using a Hybrid Rheometer (MCR302, Anton Paar, Austria) equipped with a parallel plate geometry (29). The diameters, gap, angular frequency, strain, and temperature of the parallel plate were 40 mm, 1 mm, 10 rad/s, 1%, and 25°C, respectively. The apparent viscosity curves were measured under an increasing shear rate region from 1 to 100 s^−1^.

### Antioxidant activity

#### ABTS radical scavenging activity

The antioxidant activity of HCBP was estimated by the scavenging activities of ABTS according to the pervious method with slight modifications (30). All HCBP samples with different concentrations (0.5–6.0 mg/mL) were mixed with ABTS reaction solution, and the absorbance was measured at 734 nm after 6 min reaction at room temperature under dark conditions. The ABTS radical scavenging activities (%) was calculated using the following equation:


$$\begin{array}{l} \text{Scavenging activity }=\;\left[1-\frac{\left(\text{A}-{\text{A}}_{\text{i}}\right)}{{\text{A}}_{0}}\right]\;\times 100\text{\%} \end{array}$$

where A is the absorbance of the sample, A_i_ is the absorbance of control group, A_0_ is the absorbance of blank group.

#### Ferric reducing assay power (FRAP)

Ferric reducing antioxidant power was determined as described by previous research with minor modifications (31). This method is based on antioxidants reduce a ferric complex (Fe^3+^-TPTZ) to the bule ferrous form (Fe^2+^-TPTZ) under acidic conditions. All HCBP samples with different temperature and concentrations were prepared and analyzed. The FRAP of HCBP samples was calculated by calibration curves, and the calibration equation was using Trolox as standard *y* = 0.0032x−0.0122 (*R*^2^ = 0.9996), where y was the absorbance, x was the concentration of Trolox. Analyses were performed in triplicate, and the results of FRAP in HCBP were expressed in Trolox /mL.

### Statistical analysis

All data were presented as mean ± standard, and statistical significance (*p* < 0.05) was calculated by one-way analysis of variance (ANOVA) using SPSS22.0. Origin (version 2022) was used to plot the pictures.

## Results and discussion

### Yield of HCBP

In the preparation of HCBP, yield is an important parameter to evaluate the extraction efficiency. In the present study, HCBP yield ranged from 4.56 to 7.42%, which indicated the conditions, including solid-liquid ratio, SWE extraction temperature and extraction time, had significant influence on the HCBP yield.

**Figure 1:**
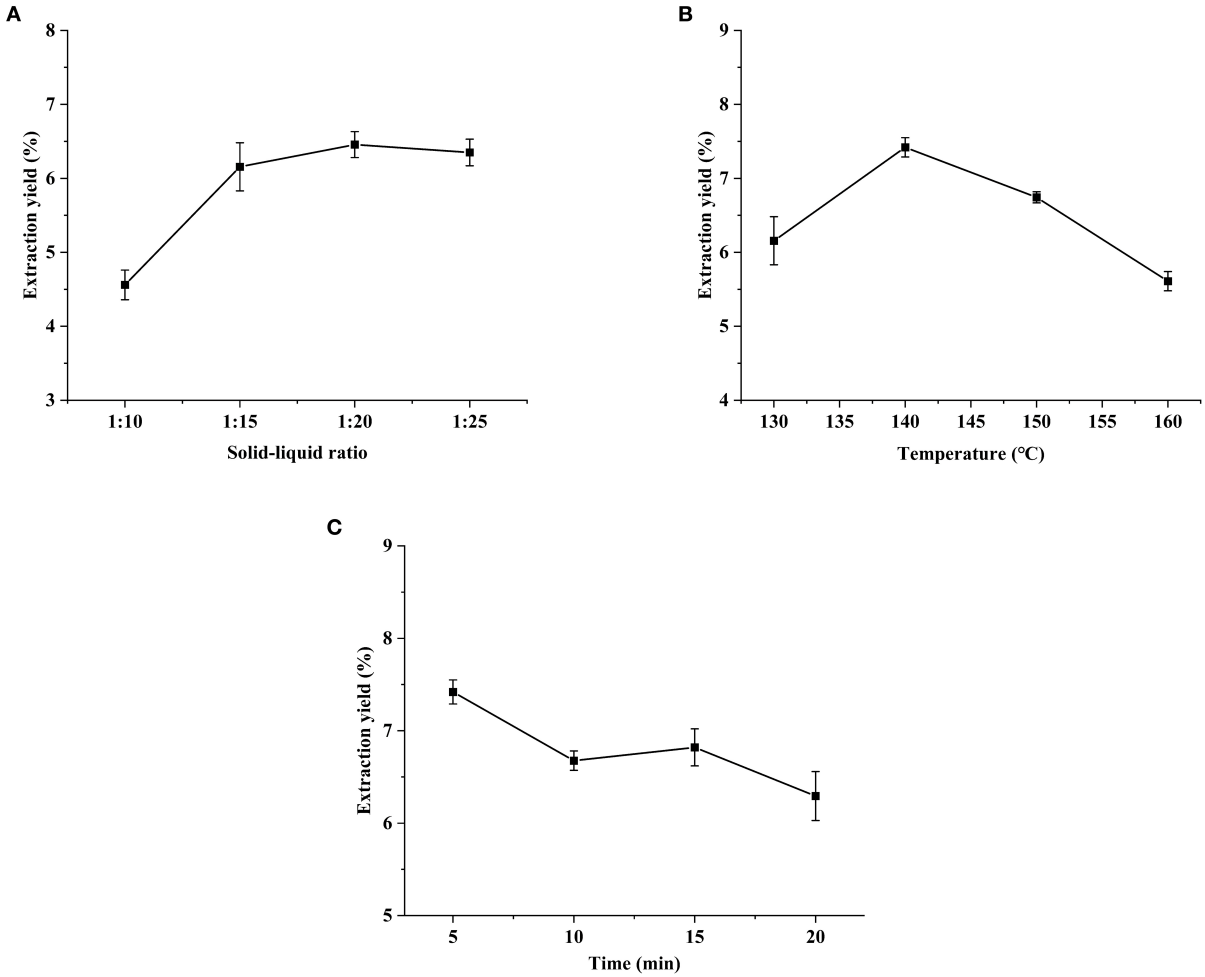
Effects of solid-liquid ratio **(A)**, extraction temperature **(B)** and extraction time **(C)** on extraction yield of HCBP.

The optimal solid-liquid ratio in the process of the preparation of HCBP was investigated. The yield of HCBP reached the optimum when the solid-liquid ratio was 1:15, the yield did not increase significantly with the increase of the solid-liquid ratio (Figure 1A). Therefore, 1:15 was selected for subsequent experiments.

The temperature was studied from 130 to 160°C. In the Figure 1B, the yield of HCBP increased and then decreased with increasing extraction temperature, and the maximum yield was achieved at 140°C. We speculated that the extraction ability of subcritical water on HCBP was relatively weak at lower extraction temperature (<140°C), while the extraction ability of subcritical water on HCBP was effectively enhanced with the increase of extraction temperature. The extraction yield of 140°C was the highest, which may be due to the balance between the relative strong extraction ability and mild degradation level of HCBP extracted by SWE. However, when the extraction temperature was higher than 140°C, the degradation of HCBP by subcritical water was stronger than that by extraction, resulting in a sharp decrease in HCBP yield with increasing extraction temperature (19). Therefore, 140°C was selected for subsequent experiments.

The extraction time was studied from 5 to 20 min. As shown in Figure 1C, the yield of HCBP decreased with the increasing extraction time, and the maximum yield of HCBP was achieved at 5 min. This may be due to the strong degradation of HCBP by subcritical water at extraction time longer than 5 min. A sharp decrease in the yield of HCBP was observed beyond this threshold.

Based on the results about the yield of HCBP, the optimal extraction conditions of subcritical water are as follows: solid-liquid ratio 1:15, extraction temperature 140°C and extraction time 5 min. These results indicate SWE can be used as a rapid and efficient method for polysaccharide extraction.

### Chemical composition of HCBP with different extraction temperatures

#### Neutral glucose

**Figure 2:**
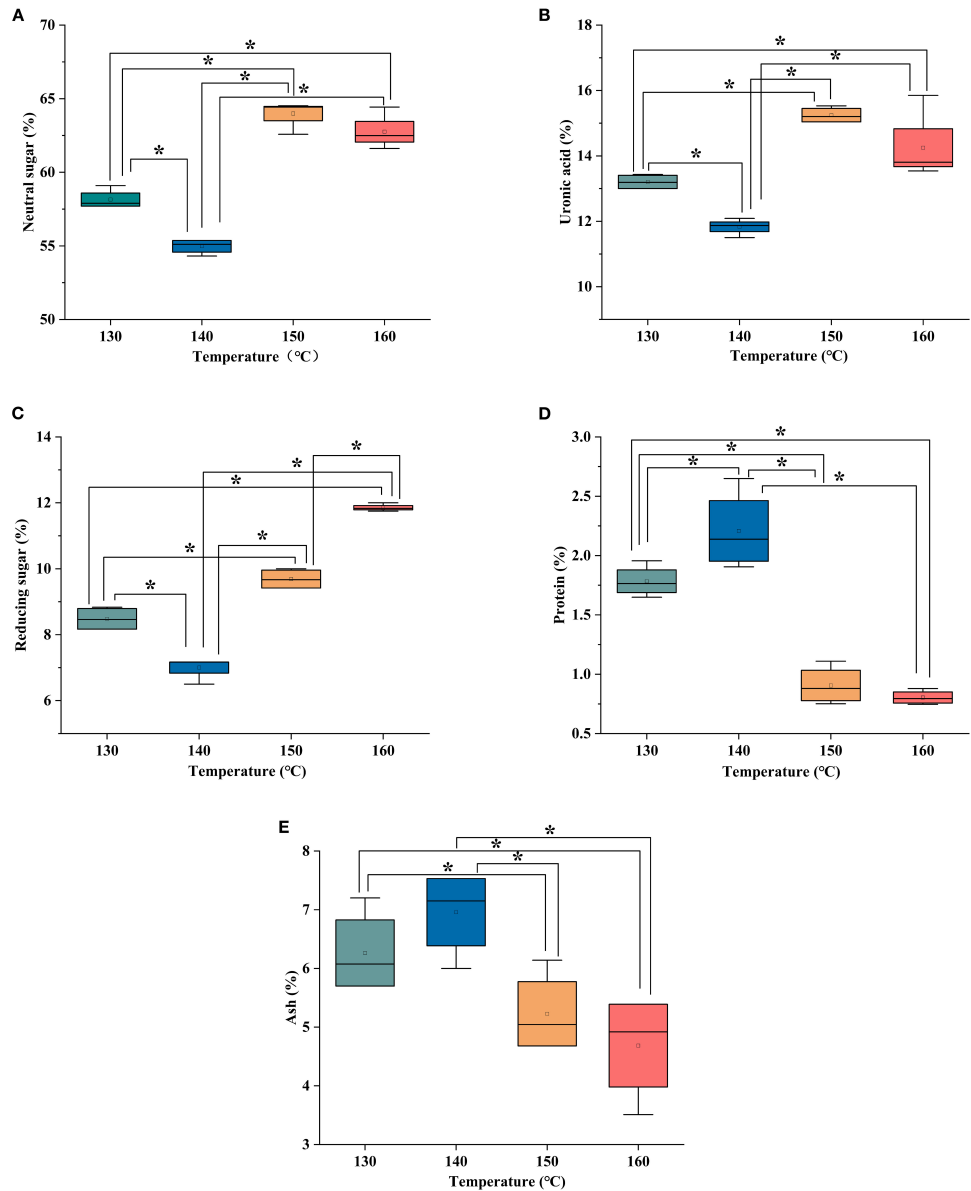
The content of neutral sugar **(A)**, uronic acid **(B)**, reducing sugar **(C)**, protein **(D)**, and ash **(E)** of HCBP by subcritical water at different temperatures, *p < 0.05.

Neutral sugar of HCBP was determined with anthrone-sulfuric acid method. The neutral sugar content was calculated using calibration curves, and the calibration equation was using glucose as standard y= 0.0032x-0.0122, where y was the absorbance, x was the concentration of glucose, and the correlation coefficient (*R*^2^) was 0.9988. HCBP extracted by different extraction temperatures were a heteropolysaccharide, the content range of neutral sugar was from 58.17 to 68.27% (Figure 2A). The neutral sugar of HCBP was the highest at 150°C and the lowest at 140°C, and all HCBP samples mainly composed of neutral sugar.

#### Uronic acid

Uronic acid of HCBP was estimated in carbazole method. The content of uronic acid in HCBP was calculated using the standard curve y= 0.0062x +0.0062 with a correlation coefficient of 0.9994, where y was the absorbance, x was the concentration of D-galacturonic acid. Figure 2B showed that the content of uronic acid in HCBP extracted at different extraction temperatures ranged from 11.05% to 15.31%, and the lowest content at 140°C and the highest content at 150°C.

#### Reducing sugar

Reducing sugar of HCBP was conducted using the 3,5-dinitrosalicylic acid (DNS) method. The reducing sugar content of HCBP was calculated using calibration curves, and the calibration equation was using glucose as standard y= 0.0012x-0.028, where y was the absorbance, x was the concentration of glucose, and the correlation coefficient was 0.9989. In the Figure 2C, the content of reducing sugar is the highest at 160°C and the lowest at 140°C, and the reducing sugar content of HCBP extracted at different extraction temperatures ranged from 6.94 to 11.86%.

#### Protein

Protein content of HCBP was measured using the Bradford method. The protein content was calculated using calibration curves, and the calibration equation was using bovine serum albumin as standard y = 0.0039x+0.0107 with a correlation coefficient of 0.9995, where y was the absorbance, x was the concentration of bovine serum albumin. The protein content of HCBP is shown in Figure 2D, all HCBP samples extracted at different extraction temperatures were found trace quantities protein, indicating SWE had a high extraction selectivity for polysaccharide, resulting in a high purity of polysaccharide products (19).

#### Ash

As shown in Figure 2E, the content of ash in HCBP extracted at different extraction temperatures ranged from 4.45 to 6.77%. The ash content of HCBP increased first and then decreased with the increase of extraction temperature, and its change trend was consistent with the change of extraction yield. The ash in HCBP mainly came from inorganic salts that were bound with polysaccharide molecules through ionic bonds, such as magnesium, potassium, calcium, and sodium salts. Therefore, with the increase of HCBP extraction yield, the ash content would also increase. At 140°C, content of ash was the highest, which was in agreement with the maximum HCBP yield.

According to the results above, 140°C was the temperature at which the yield of polysaccharide extracted by subcritical water was the highest. However, 140°C was the temperature at which the contents of neutral sugar, uronic acid and reducing sugar were the lowest, and the contents of protein and ash were the highest. Therefore, it could be seen that under the extraction condition with the highest yield, the polysaccharide content was not necessarily the highest. Hence, the production conditions should be selected according to specific requirements in industrial production.

### Molecular weight of HCBP with different extraction temperatures

Molecular weight is an important parameter of polysaccharide, which not only reflects the length of molecular chain, but also closely related to the biological activity of polysaccharide (32, 33). The molecular weight of HCBP extracted by different extraction temperatures was determined by GPC. As shown in Table 1, when the extraction temperature increased from 130 to 150°C, the molecular weight of HCBP decreased slightly. Moreover, if the extraction temperature increased to 160°C, the molecular weight of HCBP decreased dramatically. This might be because with the increase of subcritical extraction temperature, especially after the temperature rose to 150°C, the strong thermal degradation of glycosidic bonds increased, leading to the decomposition of polysaccharide molecules into smaller parts (18).

**Table 1:** The molecular weight and monosaccharide content of HCBP samples.

***H. citrina* Borani polysaccharide**	**Molecular weight (kDa)**	**Monosaccharide content (mg/g)**								
		**Mannose**	**Rhamnose**	**Glucuronic acid**	**Galacturonic acid**	**Glucose**	**Galactose**	**Xylose**	**Arabinose**	**Fucose**
130°C	159.21	1.77 ± 0.19	3.60 ± 0.44	0.15 ± 0.02	0.24 ± 0.02	31.01 ± 3.92	8.62 ± 1.32	0.79 ± 0.22	1.19 ± 0.23	2.54 ± 0.56
140°C	134.87	1.69 ± 0.28	3.66 ± 0.16	0.17 ± 0.01	0.26 ± 0.00	28.53 ± 0.70	10.60 ± 0.87	0.94 ± 0.27	0.95 ± 0.02	2.89 ± 0.34
150°C	134.06	0.61 ± 0.09	3.35 ± 0.57	0.44 ± 0.08	0.20 ± 0.03	21.25 ± 2.68	7.76 ± 1.24	0.59 ± 0.12	0.61 ± 0.12	1.83 ± 0.26
160°C	58.92	0.59 ± 0.08	3.24 ± 0.04	0.32 ± 0.13	0.19 ± 0.04	18.76 ± 0.89	7.83 ± 0.50	0.40 ± 0.04	0.28 ± 0.03	1.77 ± 0.24

### Monosaccharide composition of HCBP with different extraction temperatures

**Figure 3:**
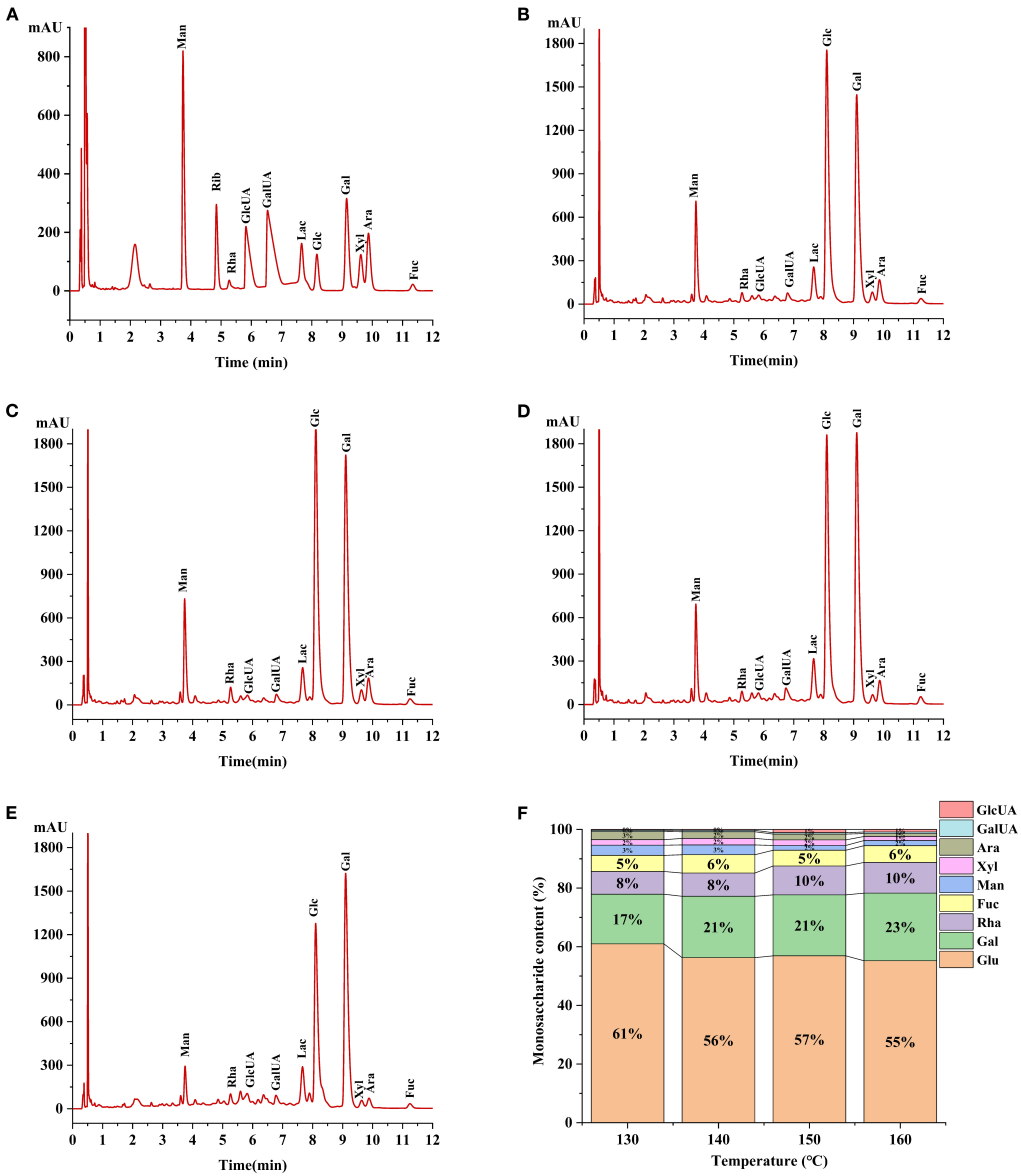
**(A)** Chromatograms of PMP derivatives of monosaccharide standard samples. **(B)** 130°C HCBP sample hydrolysate. **(C)**140°C HCBP sample hydrolysate. **(D)** 150°C HCBP sample hydrolysate. **(E)** 160°C HCBP sample hydrolysate. **(F)** The ratio of monosaccharides of HCBP at different extraction temperatures.

The monosaccharide composition plays an important role in the structural characterization and bioactive studies of polysaccharide (34). Monosaccharide analysis revealed HCBP consisted of heteropolysaccharide (Figure 3), nine monosaccharides (glucose, galactose, rhamnose, fucose, mannose, arabinose, xylose, galacturonic acid and glucuronic acid) were found in all HCBP samples with different contents. Furthermore, the monosaccharide content of HCBP at different extraction temperatures is shown in Table 1, where the mannose was 0.59–1.77 mg/g, rhamnose was 3.24–3.66 mg/g, glucuronic acid was 0.15–0.44 mg/g, galacturonic acid was 0.19–0.26 mg/g, glucose was 18.76–31.01 mg/g, galactose was 7.76–10.60 mg/g, xylose was 0.40–0.94 mg/g, arabinose was 0.28–1.19 mg/g, and fucose was 1.77–2.89 mg/g. Among them, the content of neutral sugars including glucose and galactose were the major components of HCBP samples, and the content of uronic acid including glucuronic acid and galacturonic acid were lower in HCBP samples. The results indicate that the HCBP samples are mainly neutral sugars, which is in agreement with the results of section neutral glucose.

### Spectrum analysis of HCBP with different extraction temperatures

#### Ultraviolet—visible spectrum

**Figure 4:**
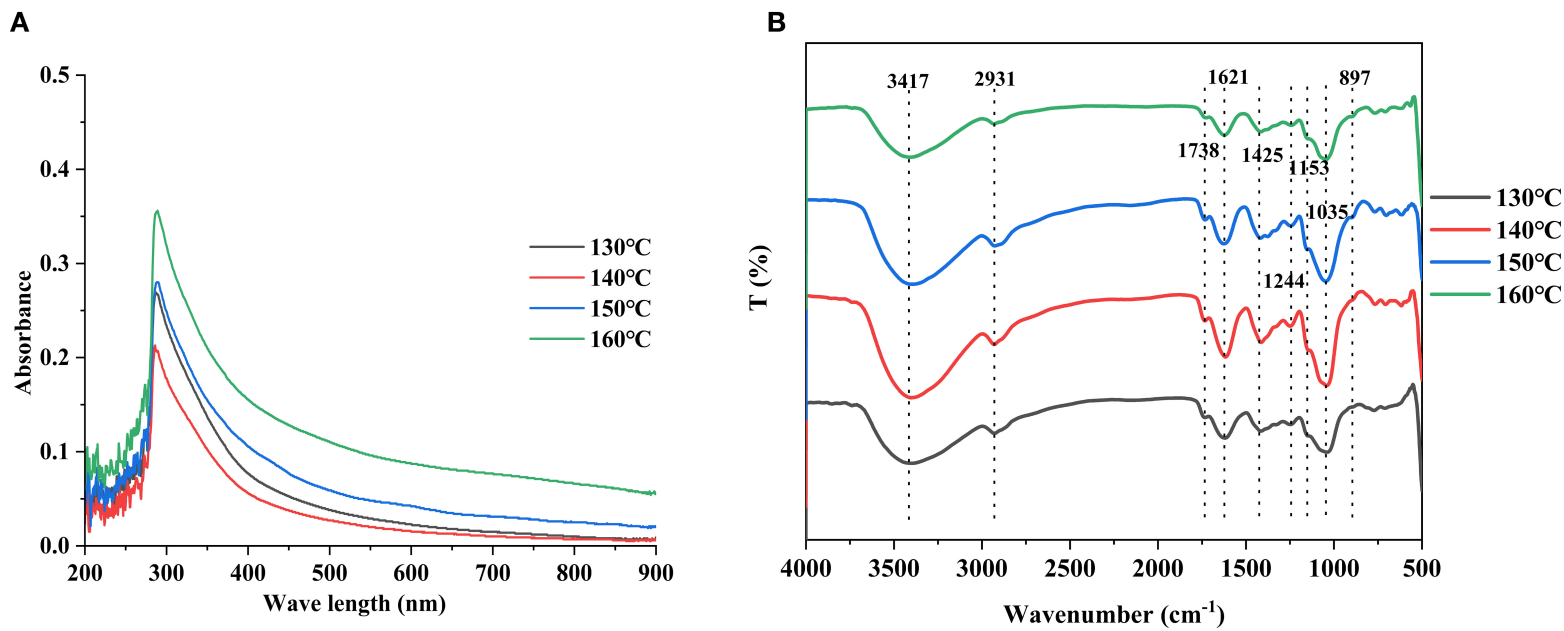
UV spectrum **(A)** and FT-IR spectra **(B)** of HCBP samples.

As shown in Figure 4A, a similar absorption ultraviolet–visible spectra of 200–900 nm were found in HCBP samples extracted at different temperatures. A weak absorption peak at 280 nm was found in all HCBP samples, which indicated the presence of a small amount of protein. In addition, no other absorption peaks indicated high purity of HCBP samples and low content of non-polysaccharide components, which was in accordance with chemical composition analysis results. However, UV spectrum showed that HCBP extracted at 160°C had the largest absorption peak, while the content of HCBP protein extracted at 140°C was the highest by Bradford's method. This difference may be caused by unknown components in HCBP extracted at different temperatures.

#### Fourier transform infrared spectrum

The IR spectrum of HCBP is shown in Figure 4B. A high level of similarity of the FT-IR absorption pattern was found between HCBP samples prepared at different temperatures. A peak at 3,417 cm^−1^ belonged to the stretching vibration of O–H, and the strong and broad peak shape was typical of carbohydrates, which indicated the presence of inter-molecular or intra-molecular hydrogen bonds (35). Moreover, the characteristic absorption at 2,931 and 1,244 cm^−1^ was ascribed to C–H stretching and bending vibrations of CH_2_ group in the sugar ring (36). The characteristic absorption at around 1,738 cm^−1^ corresponded to the vibration of esterified carboxyl (COO–CH3), and 1,621 cm^−1^ was derived from free carboxyl (COO–) groups, confirming the presence of uronic acid (37). This result was in accordance with the results of section uronic acid. Notably, the peak at 1,300–800 cm^−1^ represented the fingerprint region of polysaccharide (19). In addition, the weak bands near 895 cm^−1^ indicated the glucosyl residues in β-configurations existed in HCBP samples.

### Scanning electron microscopy analysis

**Figure 5:**
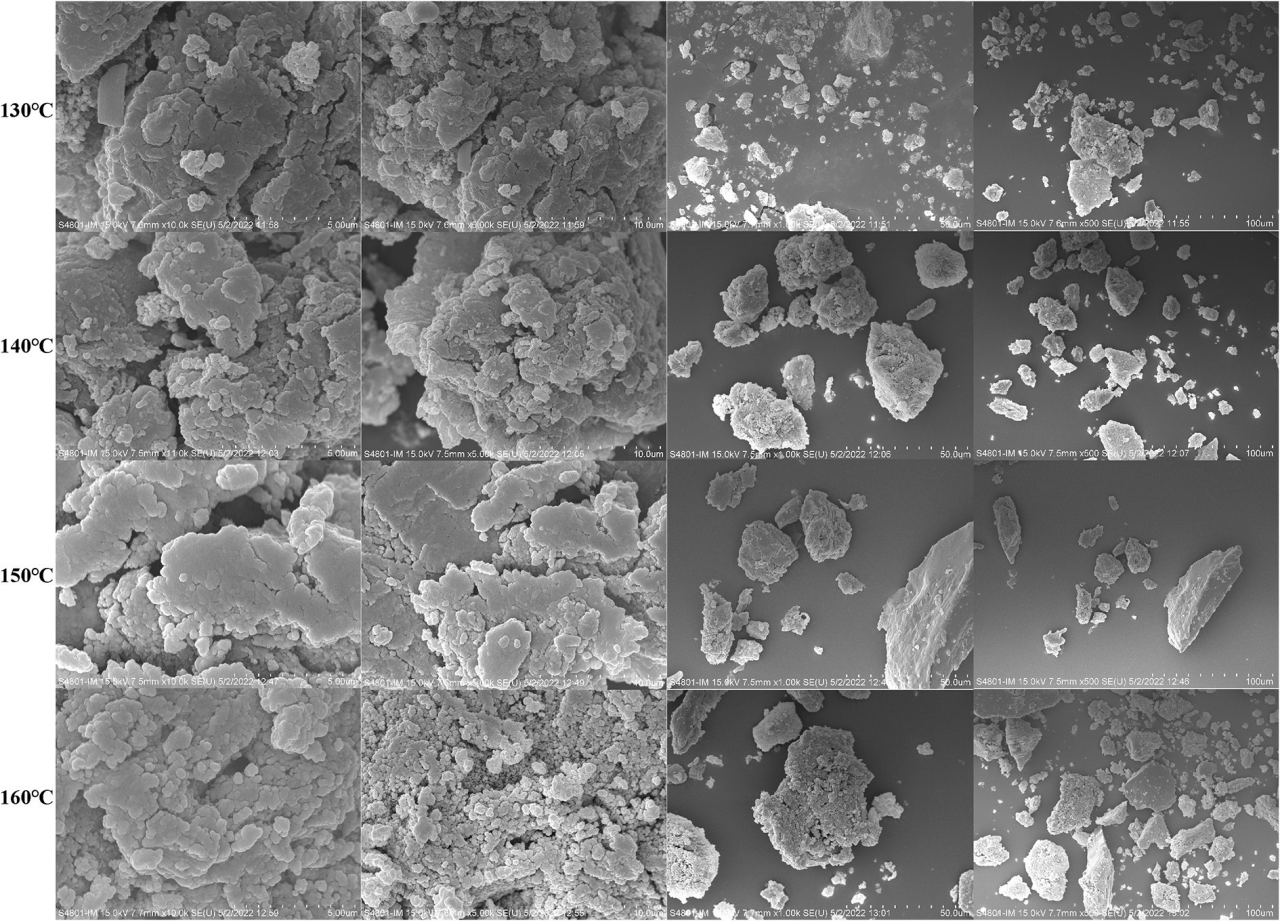
SEM images of HCBP extracted by different temperatures at 5-, 10-, 50-, and 100-μm.

Scanning electron microscopy (SEM) is a technique for observing the morphological characterization including size, shape and porosity of polysaccharide surface (38). The SEM images of HCBP samples at 5-, 10-, 50-, and 100-μm are displayed in Figure 5, and similar SEM images of the HCBP samples with different extraction temperatures were found. All HCBP samples were characterized by rough surface, obvious particle gap, loose structure, and irregular shape. This may be due to the fact that all HCBP samples were mainly composed of neutral sugar, and had less—COOH in the glycosyl, leading to less opportunities for the formation of compact high-level structure between molecules, and resulting in obvious sample gap and loose structure.

### Rheological properties of HCBP

**Figure 6:**
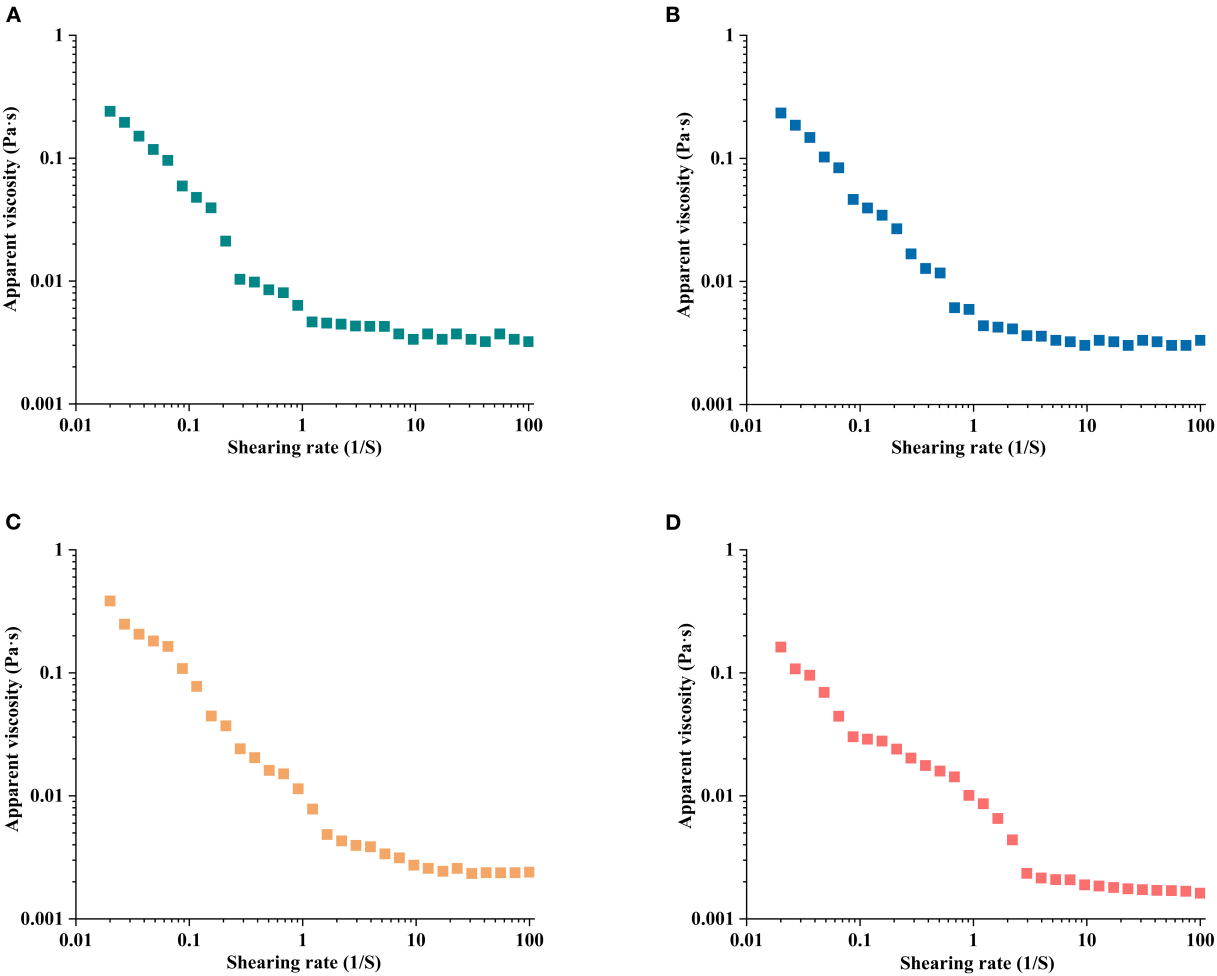
The flow behavior of HCBP by subcritical water at 130°C **(A)**, 140°C **(B)**, 150°C **(C)**, and 160°C **(D)**.

The steady shear flow curves of HCBP solutions extracted by 130, 140, 150, and 160°C are described in Figure 6. All HCBP samples displayed a similar and typical shear flow behavior. The apparent viscosity of all HCBP solutions were related to the shear rate, and it decreased obviously with the increase of shear rate. The polymer chain of HCBP flowed directionally in the flow field, resulting in shear thinning. This phenomenon of shear thinning behavior described as pseudoplastic fluid or non-Newtonian fluid (39). With the increase of shear rate, the randomly placed molecules were more aligned in the flow direction, resulting in the decrease of the interaction between adjacent molecules and gradually disentangled of polysaccharide (40). Significantly, the apparent viscosity of HCBP decreased rapidly at low shear rate (0.001–1 s^−1^) and lower temperature (130–140°C), and the apparent viscosity of HCBP remained almost constant with the increase of shear rate. However, the apparent viscosity of HCBP at higher temperature (150–160°C) still decreased at higher shear rate (1–10 s^−1^) with the increase of shear rate. Rheological behaviors of samples were strongly related to physiochemical properties of polysaccharide including molecular weight and chain stiffness (41). The changes of apparent viscosity and molecular weight of HCBP samples showed a similar downward trend with the increase of temperature. HCBP with different rheological properties could be prepared by regulating SWE conditions. The results showed that HCBP extracted by SWE could be used as a thickening agent in the food industry.

### Antioxidant activity

#### Ferric reducing assay power

**Figure 7:**
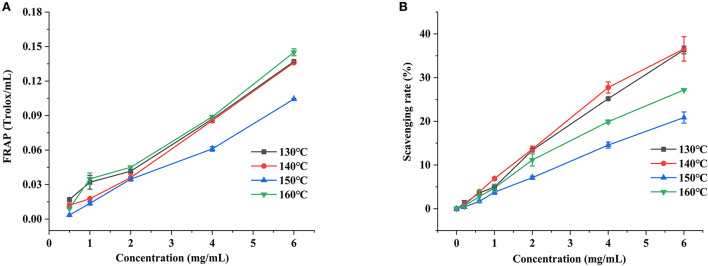
Ferric reducing assay power **(A)** and ABTS radical scavenging activity **(B)** of HCBP by subcritical water at different temperatures.

FRAP is a method used to determine the total antioxidant capacity of natural substances (42). As shown in Figure 7A, an obvious concentration-dependent at the range of 0.5–6 mg/ml for the total antioxidant capacity of FRAP in the HCBP samples was found. Remarkably, the extraction temperature also had a great influence on the FRAP of HCBP samples, especially for the samples obtained at 160°C, which performed best total antioxidant capacity of FRAP during the test. The polysaccharide extracted at 160°C had lower molecular weight, and the polysaccharide with lower molecular weight often had better antioxidant activity (43). In addition, the reducing sugar content was the highest at 160°C, which might be the reason for the optimal total antioxidant capacity of FRAP.

#### ABTS radical scavenging activity

ABTS is a non-physiological free radical used to evaluate the antioxidant power of natural products, the ABTS radical scavenging capacity of HCBP is shown in Figure 7B. The scavenging capacity of HCBP to ABTS radical increased with the increase of concentration, showing an obvious dose-dependent at the range of 0.2–6 mg/ml. Moreover, it was also clearly that the extraction temperature played a more important role at improving ABTS radical scavenging capacity, but different from total antioxidant capacity of FRAP (160°C was best total antioxidant capacity of FRAP), the polysaccharide extracted at 130 and 140°C had the higher ABTS scavenging rate. In addition, polysaccharide-protein conjugates containing hydrophobic pores and cracks enhance antioxidant activity (44). Therefore, the polysaccharide-protein conjugate might help the radical scavenging ability of HCBP. The protein content of polysaccharide extracted at 130 and 140 °C was the higher, which might be the reason for its best ABTS radical scavenging capacities.

## Conclusion

In this work, SWE was employed to extract polysaccharide from *H. citrina* Borani. The highest yield of HCBP was achieved under the following conditions: a solid–liquid ratio 1:15, extraction temperature of 140°C, and extraction time of 5 min. Then, the physicochemical properties of HCBP extracted by subcritical water at different temperatures were evaluated. The results showed that HCBP extracted at 140°C possessed the lowest contents of neutral sugar, uronic acid and reducing sugar, and the highest contents of protein and ash. HCBP extracted at 150°C had the highest contents of neutral sugar, uronic acid. HPLC revealed all HCBP samples were mainly composed nine monosaccharides with different proportions. GPC showed that the molecular weight of HCBP samples decreased with increasing temperature. UV confirmed that HCBP samples contained a small amount of protein, and FT-IR revealed that the glycosidic bond of HCBP samples were β-configuration. The apparent viscosity of HCBP at higher temperature (150–160°C) decreased with the increase of shear rate (1–10 s^−1^), but nearly constant at lower temperature (130–140°C). The HCBP samples had the characteristics of rough surface, obvious particle gap, loose structure and irregular shape observed by SEM. HCBP had good antioxidant effect, among which, the FRAP activity of HCBP extracted at 160°C was the strongest, and the ABTS radical scavenging activity of HCBP extracted at 130 and 140°C was better. In conclusion, our study indicates that the extraction condition of the highest yield may not the optimum condition of polysaccharide content and antioxidant activity. Hence, the production conditions should be selected according to specific requirements. In addition, this study suggests that HCBP extracted by SWE could provide an inexpensive raw material for the development of food thickening agent and natural antioxidants, increase the value of *H. citrina* Borani products and promote its industrial development.

## Data availability statement

The raw data supporting the conclusions of this article will be made available by the authors, without undue reservation.

## Author contributions

YT: investigation, conceptualization, formal analysis, data curation, writing—original draft, and writing—review and editing. YZ: investigation, formal analysis, and data curation. YB: investigation and data curation. XW: investigation, visualization, and software. YH and XW: investigation. ZS: investigation, methodology, formal analysis, funding acquisition, supervision, and writing—review and editing. All authors contributed to the article and approved the submitted version.

## Conflict of interest

The authors declare that the research was conducted in the absence of any commercial or financial relationships that could be construed as a potential conflict of interest.

## Publisher's note

All claims expressed in this article are solely those of the authors and do not necessarily represent those of their affiliated organizations, or those of the publisher, the editors and the reviewers. Any product that may be evaluated in this article, or claim that may be made by its manufacturer, is not guaranteed or endorsed by the publisher.
